# Using Stimulus-Equivalence Technology to Teach Skills About Nutritional Content

**DOI:** 10.1007/s40614-020-00250-2

**Published:** 2020-04-21

**Authors:** Erik Arntzen, Jon Magnus Eilertsen

**Affiliations:** Department of Behavioral Science, Oslo Metropolitan University, PO Box 4, St. Olavs Plass, 0130 Oslo, Norway

**Keywords:** Carbohydrates, College students, Effectiveness, Nutrition, Stimulus equivalence

## Abstract

Twenty-two adult participants, assigned to three conditions, were trained nutrition knowledge (i.e., carbohydrate values) for different food items. In a stimulus sorting test, the participants were asked to sort stimuli (names of food items) into one of three different ranges of carbohydrate values ("less than 20", "20–40", "more than 40" gram per 100 gram). Conditional-discrimination training and testing followed the sorting test, and finally, a postclass formation sorting test of the stimuli used in the conditional-discrimination training. The conditional-discrimination training used tailored stimuli, that is, the food items that each of the participants categorized incorrectly in the sorting test. Participants exposed to Conditions 1 and 2 were trained on six conditional discriminations and tested for the formation of three 3-member classes. Conditions 2 and 3 had a “don’t know” option together with the three different ranges of carbohydrate values in the sorting for tailoring the stimuli. Participants exposed to Condition 3 trained were trained on 12 conditional discriminations and tested for the formation of three 5-member classes. The main findings showed that all but one of the participants responded correctly on at least one test for equivalence class formation and sorted the stimuli correctly in the postclass formation sorting test.

It is common to read information stating that food choice affects health, and that proper nutrition is related to a healthy lifestyle. Hence, to make these healthy food choices, nutritional knowledge is a prerequisite (Grunert et al., 2012). In particular, several authors have pointed out the importance of knowledge about nutrition as one of the factors for having good health (e.g., Lessa, Cortes, Frigola, & Esteve, 2017; Miller & Cassady, 2015; Wardle, Parmenter, & Waller, 2000). Furthermore, studies have shown a correlation between mother’s knowledge about nutrition and healthy weights for their children (Yabancı, Kısaç, & Karakuş, 2014), and less knowledge about nutrition was correlated with less compliance with the dietary guidelines for many food groups (Vereecken & Maes, 2010).

A series of studies employing nutrition education have been shown to be effective in teaching knowledge about nutrition in adults (Allen, Smith Taylor, & Kuiper, 2007; Clifford, Anderson, Auld, & Champ, 2009; Tessaro, Rye, Parker, Mangone, & McCrone, 2010) and in children (Pears et al., 2012). For example, Allen et al. (2007) found that a 30-min session with information about nutrition influenced the choice of food in a simulated fast-food environment. Tessaro et al. (2010) used a computer-based interactive intervention (Cookin’ Up Health) to teach women from a rural district knowledge about nutrition.

Nastally, Dixon, McKeel, and Fleming (2010) have argued that it is not clear what is the most efficient way to arrange procedures to increase knowledge about nutrition; strategies so far have included a variety of procedures, such as information about nutrition labels and food exposures. One such strategy that has been shown to be useful in teaching different skills in a variety of participants are procedures based on stimulus-equivalence technology, known for more than 30 years. Later, the term “equivalence-based instruction” (EBI) has been used (Fienup, Covey, & Critchfield, 2010). Examples of procedures based on stimulus-equivalence technology or EBI are summarized as follows: (1) establishing different skills in college students, such as (a) neuroanatomy (Fienup et al., 2010; Fienup, Mylan, Brodsky, & Pytte, 2016; Pytte & Fienup, 2012; Reyes-Giordano & Fienup, 2015), (b) statistical inference (Critchfield & Fienup, 2010; Fields et al., 2009; Fienup & Critchfield, 2010, 2011; Fienup, Critchfield, & Covey, 2009), (c) trigonometric relations (Ninness et al., 2009), and (d) intraverbals (Walker, Rehfeldt, & Ninness, 2010); (2) establishing different skills in adults with brain injury (e.g., Cowley, Green, & Braunling-McMorrow, 1992; Guercio, Podolska-Schroeder, & Rehfeldt, 2004); (3) establishing different skills in typically developing children (Lynch & Cuvo, 1995); (4) establishing different skills in people with autism spectrum disorder (ASD; e.g., Arntzen, Halstadtro, Bjerke, & Halstadtro, 2010; Arntzen, Halstadtro, Bjerke, Wittner, & Kristiansen, 2014; Stanley, Belisle, & Dixon, 2018).

One line of research on healthy food choices has focused on how to influence participants' preferences. When studying food preference in children, procedures based on stimulus-equivalence technology have been used to investigate the formation of preference for brands of food and drinks (dos Santos & de Rose, 2018, 2019) and the evaluation of food (Straatmann, Almeida, & Rose, 2014). Additionally, procedures based on stimulus-equivalence technology have been used to make accurate portion size estimations among college students (Hausman, Borrero, Fisher, & Kahng, 2014) and children (Hausman, Borrero, Fisher, & Kahng, 2017).

Instead of manipulating food preferences, it is possible to teach participants the nutritional content of different food items. Nastally et al. (2010) exposed six college students to different tasks, including assessment of the caloric content of different food items. The participants were exposed to pre- and posttests for food preferences, pre- and posttests for the caloric content of different food items and to conditional-discrimination training with pictures of the food items from the second pretest. In the pretest for the caloric content, the participants were asked to sort pictures of 18 food items according to three categories of caloric content. These category cards were “Under 500 Calories,” “500–1,000 Calories,” and “Over 1,000 Calories.” The participants were asked to name the food items and sort the items according to these categories. The stimuli mislabeled in the sorting test were used in the conditional-discrimination training. If there were not enough stimuli mislabeled, the experimenter selected a food item randomly. However, there was no information reported on how many times the experimenter had to pick a food item. Thus, whether any of the classes were partly formed before the conditional-discrimination training is not available to the reader. The conditional-discrimination training was arranged as two one-to-many (OTM) training structures. The first OTM training structure was performed with the category cards as A stimuli. The procedure started with training AB relations, followed by AC relations and a mix of AB and AC relations. The participants were then tested for the emergence of BC and CB relations in two 18-trial blocks. After a short break, the participants were exposed to a second OTM training with CD and CE relations arranged as the AB and AC training. This training was followed by testing for emergent relations of BC and CB relations in two 18-trial blocks, respectively. The results showed that training necessary conditional discriminations and testing emergent relations improved the nutrition labeling skills in all six participants. After the conditional-discrimination training, they found that participants were able to partition the stimuli according to the respective caloric content. Additionally, more than half of the participants made a healthier food choice after the training and testing.

Nastally et al. (2010) called for experiments with measurements of nutrition other than the caloric content of food items, such as carbohydrate levels of food items, which is also in accordance with the fact that several individuals are on a low-carb diet and may find that information more useful. Thus, it seems essential to determine whether conditional-discrimination training and testing for emergent relations with nutrition information about carbohydrates is as effective as shown in Nastally et al.

Most food items contain carbohydrates, such as fruits, grains and soft drinks, whereas others, such as different meat products and fish, do not (Norwegian Food Safety Authority, 2019). It is common knowledge that bread, pasta, beans, and potatoes are carbohydrate-rich foods, but not the precise quantity of carbohydrates per 100 gram found in these foods is not well known. For example, it is not apparent that raisins and pretzel sticks have more than 40 g of carbohydrates per 100 g. Therefore, it could be useful to introduce a “don’t know” option in a sorting test.

The overall goal of the present experiment was to employ procedures based on stimulus-equivalence technology to train knowledge about nutritional content (e.g., carbohydrate levels) in adult participants. To show the efficacy of an such a technology, it is essential to show how the approach is useful not only to small classes (names of food items) but also to larger classes (names of food items). Thus, the experiment includes three conditions in which participants were exposed to 6 or 12 conditional discriminations and were tested for the emergence of smaller classes (three 3-member classes) or larger classes (three 5-member classes). Additionally, we wanted to show the importance of tailoring stimuli for each participant. Therefore, the participants categorized the stimuli with name of different food items according to three levels of carbohydrate values ("less than 20", "20–40", "more than 40" gram per 100 gram) in a sorting test. The stimuli used in the conditional-discrimination training were stimuli that each participant did not categorize correctly according to the carbohydrate values. Finally, to explore whether the performance was maintained in a testing format other than matching-to-format (MTS), a postclass formation sorting test followed the conditional-discrimination training and testing.

## Method

### Participants

Twenty-two adults affiliated with Karlstad University and personal contacts participated in the experiment. Two more participants withdrew from the experiment, and their data were not included. The ages of the participants were between 19 and 54, with the mean age of 26 years. They were informed that the experiment was within the field of learning psychology. Participation in the experiment was compensated with 200 Swedish kroner (approximately US$21.40). Each participant was quasi randomly assigned to one out of three conditions, with eight participants in Conditions 1 and 2, respectively, and six participants in Condition 3. The participants had to read a consent form upon arrival. The form stated who was conducting the experiment and that they would remain anonymous. Also, they were informed about their rights to withdraw from the experiment at any given time. Finally, the participants were fully debriefed at the end of the experiment.

### Apparatus and Setting

The experiment was run in a quiet room at Karlstad University. The room was approximately 6 m^2^ and equipped with two chairs and a table. An HP ProBook 470 GP laptop computer running Windows 10 64-bit. The screen was 17.4 in. Participants used an external mouse to click on the stimuli. Custom-made software arranged and controlled the presentation of trials. The software recorded the order of the presented trials, the number of training and test trials, the reaction time to sample and comparison stimuli, correct/incorrect comparison choices, the delivery of programmed consequences, the duration of the experiment, and a summary of a participant’s performance on an equivalence test.

### Stimuli

Table 1 shows the stimuli used in the conditional-discrimination training and testing. The stimuli consisted of three ranges of carbohydrate values and the printed names of different food items. The food items were selected based on their carbohydrate content (Norwegian Food Safety Authority, 2019). The 21 stimuli in the top section of Table 1 were used for Conditions 1 and 2. All 33 stimuli were used for Condition 3. The carbohydrate range cards read “less than 20,” “20–40,” and “more than 40,” except for two participants, P13500 and P13501, who had range cards that read “0–20,” “20–40,” and “40–80.” Additionally, due to a procedural error, these two participants were shown 18 instead of 21 stimuli. In Conditions 2 and 3, a stimulus card with the text “don’t know” was added to the three carbohydrate ranges. The size of the cards varied from 3 cm × 3 cm to 5 cm × 5 cm.

**Table 1:** Overview of the stimuli used in all three conditions

1	2	3
Less than 20	20–40	More than 40
Potatoes	Cashew nuts	Popcorn
Garlic, raw	Ketchup	Quinoa
Grapes	Tomato paste	Raisins
Bananas	Couscous	Garlic powder
Peanut butter	Dark chocolate 70 %	White pepper
Carrots	Big One Classic	Axa fruit muesli
Raspberries	Boiled pasta	Oatmeal
Fish fingers	Taco spice mix	Taco shells
Blueberries	Boiled basmati rice	Polar bread
Brie	Frozen pommes frites	Crispbread, Wasa, Husman
Nestea Iced Tea	Pancakes	Pretzel sticks

*Note*. The 21 stimuli above the dashed line were employed for Conditions 1 and 2, respectively. All 33 stimuli were employed for Condition 3. The value for Quinoa is based for raw Quinoa

### Design and Dependent Measures

The design is a demonstration of how participants learned carbohydrate levels when exposed to a procedure based on stimulus-equivalence technology. A sorting test is used to tailor the stimuli in the experiment. A postclass formation sorting test is used to study how the participants’ class formation is maintained in a different test format. Dependent variables were the stimuli that were incorrectly grouped in the sorting test, trials to mastery criterion in the conditional-discrimination training, the percent correct responses during the test for emergent relations and the number of stimuli correctly grouped in the postclass formation test.

### Procedure

Four different phases—sorting and tailoring of stimuli, conditional discrimination-training, and testing, and postclass formation sorting—were employed in the present experiment.

#### Phase 1: Sorting and Tailoring of Stimuli

After signing the consent form, the participants were handed a deck of cards containing all of the printed food items. For participants in Conditions 1 and 2, the deck consisted of 21 printed food items, and in Condition 3, the deck consisted of 33 printed food items. The carbohydrate ranges were “less than 20,” “20–40,” and “more than 40.”

The category names with carbohydrate ranges and the names of the different food items were presented on laminated plastic cards. The names were written in printed black text on a white background. Because we wanted the participants to group the food items according to the categories, the sorting was arranged as a table-top procedure. The stimulus cards with the labels of carbohydrate ranges were placed in front of the participants. The instructions to the participants were as follows: “On the table you will see 3 (4) category labels which indicate carbohydrate amounts per 100 g of each for the food items. Your task is to sort the food items into the categories you think they belong to.” For Conditions 2 and 3, the participants were told that they could also sort the stimuli in the “don’t know” category. The participants were told to let the experimenter know if they did not know the food item. The only food item some of the participants were uncertain about, was “Big One Classic.” The experimenter told the participants that it was a frozen pizza. The experimenter left the room while the participants sorted the cards.

When the participants had sorted the cards, they were told to leave the room for a few minutes while the experimenter assessed the food items they had sorted incorrectly. The incorrectly sorted food items were used as stimuli in the conditional-discrimination training and testing. As a result, for each participant, the stimuli were individualized or tailored in training and testing. For participants in Conditions 1 and 2, two incorrectly sorted food items from each category were randomly chosen and used as stimuli B1 and C1, B2 and C2, B3, and C3. The categories “less than 20”, “20–40”, and “more than 40” were used as stimuli A1, A2, and A3, respectively. For the participants in Condition 3, six more incorrectly sorted food items were selected, D1, D2, D3, E1, E2, and E3. If in the Conditions 1 and 2, and Condition 3 participants did not make six or 12 incorrect sorting responses, respectively, the remaining stimuli were randomly chosen. Consequently, the experimenter had to choose one stimulus each for four participants. The stimuli were then loaded into the customized software and used during the conditional-discrimination training and testing and postclass formation sorting test.

#### Phase 2: Conditional-Discrimination Training

The participants were seated in front of the computer, and the computer screen displayed the following written instructions:

Thank you for participating in the experiment. It is an experiment in learning psychology and requires no prior computer-knowledge. In short, you should click some stimuli that appear on the screen. The goal is to obtain as many correct choices as possible. When you move the mouse cursor onto the stimulus in the middle of the screen and click it, more stimuli will appear in the corners. Mouse clicks on one of the correct stimuli in the corners will be followed by presentation of the text “Correct” or similar. Clicking on one of the incorrect stimuli will be followed by the text “Wrong.” This is how you will find out whether your response was correct and incorrect. After a while, you will not be notified if your response is correct or incorrect; no text will appear on the screen. Click start to begin the experiment.

When the participants clicked the start button, a stimulus appeared in the middle of the screen, and three other stimuli appeared in the corners of the screen. One corner remained blank, and the location of the blank corner was randomized. When the participant clicked one of the three stimuli in the corners, all stimuli disappeared, and programmed consequences were presented in the middle of the screen. If the participants clicked the stimulus defined as correct, words such as “Awesome,” “Correct,” and so on was presented. If they clicked the stimulus defined as incorrect, the word “Wrong” was presented. The programmed consequence was presented for 500 ms and was followed by an intertrial interval (ITI) of 500 ms.

An OTM training structure was used to train the necessary conditional discriminations. The trials were presented on a concurrent basis, which means that for Conditions 1 and 2, AB and AC trials were mixed from the beginning of the training, whereas in Condition 3, AB, AC, AD, and AE trials were mixed from the beginning of the training. The baseline relations were presented in blocks of 30 trials for participants in Conditions 1 and 2 (see Table 2). For participants in Condition 3, the baseline relations were presented in blocks of 60 trials. A mastery criterion of 95% was required to proceed throughout the training blocks. The programmed consequences were presented for every trial until the participants met the mastery criterion in a block. Then, the programmed consequences were thinned in blocks from 75% to 50%, and 0% as long as the participants met the mastery criterion in each block before the test phase for emergent relations. The number of training blocks required to reach the test varied across participants based on their performance.

**Table 2:** Overview of parameters for trained and tested relations

	Relations and trials per block	Mastery criterion	Probability of programmed consequences (%)
Conditions 1 and 2			
Training OTM	30 AB and AC trials	96.7%	100, 75, 50, 0
Testing	54 Baseline, Symmetry, and Equivalence trials	Min of 94.4% correct of each relation type	0
	AB, AC, BA, CA, CB, BC		
Condition 3			
Training OTM	AB, AC, AD, AE	95%	100, 75, 50, 0
Testing	180 Baseline, Symmetry, and Equivalence trials	Min of 94.4% correct of each relation type	0
	AB, AC, AD, AE, BA, CA, DA, EA, BC		
	BC, BD, BE, CB, CD, CE, DB, DC, DE		
	EB, EC, ED		

#### Phase 3: Test for Emergent Relations

The two test blocks consisted each of 54 trials for Conditions 1 and 2, and 180 trials for Condition 3. (See Table 2 for a detailed overview of the training and testing parameters, and the trained and tested relations.) The 54-trial block included 18 baseline (AB and AC), 18 symmetry (BA and CA), and 18 equivalence (BC and CB) trials. The criterion for responding in accordance with stimulus equivalence was a minimum of 17 of 18 correct trials (94.4%). The 180-trial block included 36 baseline (AB, AC, AD, and AE), 36 symmetry (BA, CA, DA, and EA), and 108 equivalence (BC, CB, BD, DB, BE, EB, CE, EC, DE, and ED) trials. The criterion for responding in accordance with stimulus equivalence was a minimum of 34 of 36 baseline and symmetry trials and 102 of 108 equivalence trials (94.4%). All trials in the test blocks were presented under extinction conditions with no programmed consequences.

#### Phase 4: Postclass Formation Sorting

When the participants had completed the test for emergent relations, the written text “congratulations, you can now contact the experimenter” appeared on the screen. The participants were asked to briefly leave the room while the experimenter (the second author) arranged the postclass formation sorting. The sorting test was done with customized software (and not arranged as a table-top test as in the sorting test) on the same laptop computer that administered the conditional-discrimination training and testing. The participants were seated in front of the computer, and the computer screen displayed the following written instructions:

Sort the pictures as you want. When you have sorted the pictures as you want, please mark the sorting by holding down the left mouse button and draw by moving the mouse. The stimuli will be placed on top of each other; you will have to drag them to any other location on the screen. By moving one of the stimuli, you can undo the drawn markings.

All of the stimuli used during the conditional-discrimination training and testing were presented on top of each other in a stack. The order of the stimuli in the stack was randomized across presentations. The participants were told that the sorting test would be presented twice, and then the experiment was finished.

### Interobserver Agreement

The experimenter and another trained person scored whether the stimuli used in the conditional-discrimination training and testing for emergent relations were the stimuli that the participants did not categorize correctly in the sorting test. Thirty percent of all cases were scored. The interobserver agreement was scored as agreement/total number of cases × 100 (Kazdin, 1982) and was found to be 96.6%.

## Results

### Sorting and Tailoring of Stimuli

**Fig. 1:**
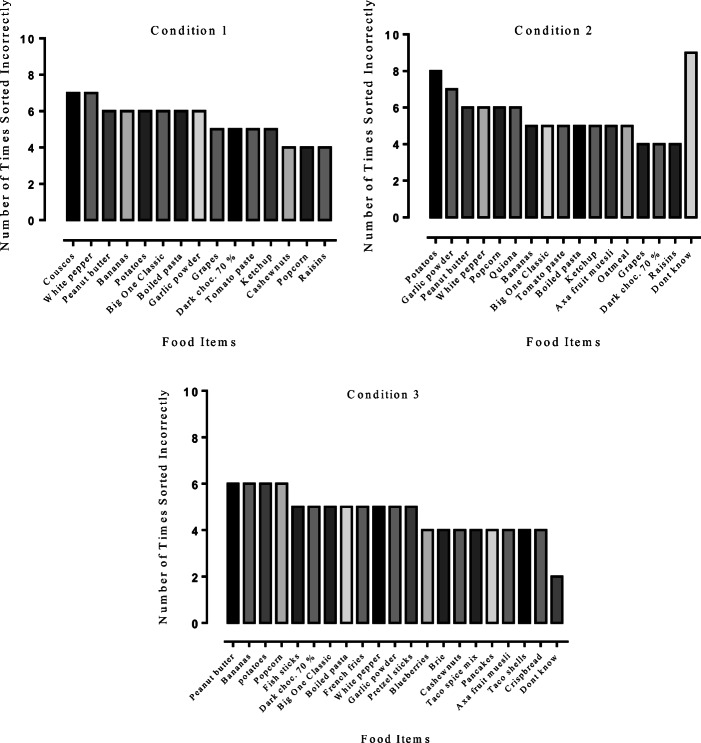
Total number of times the different food items were sorted incorrectly for Conditions 1, 2, and 3. Food items sorted incorrectly fewer than four times are not included in the figure

**Fig. 2:**
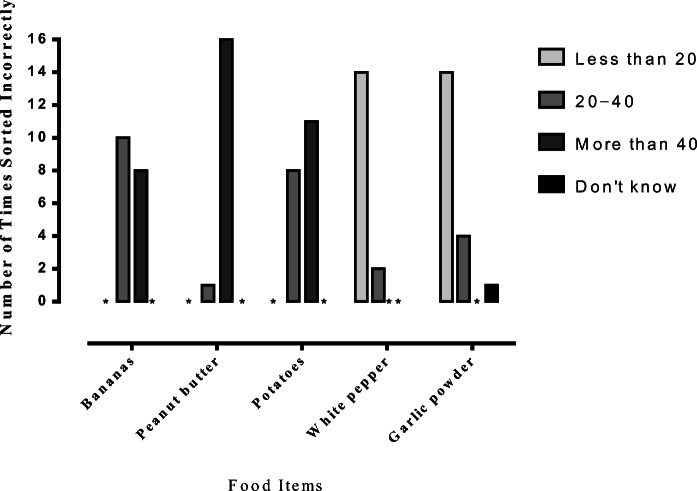
Five food items were ranked as the top three incorrectly sorted items. *Notes.* * = no participant sorted the item at that specific carbohydrate range

**Fig. 3:**
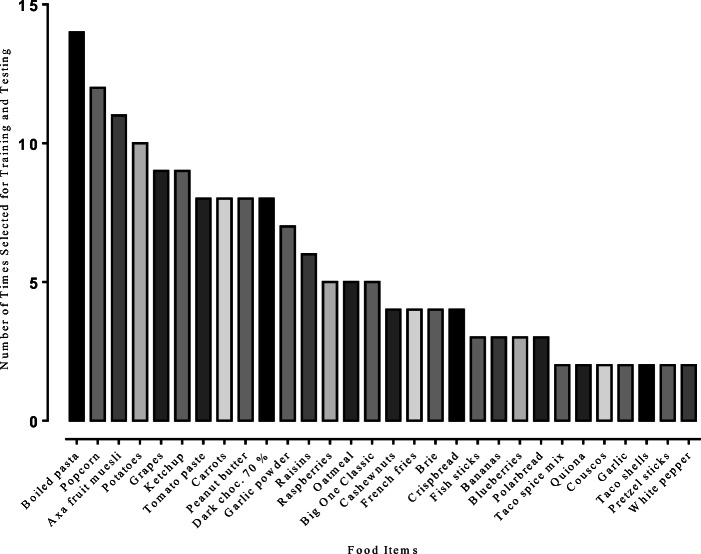
Number of times different stimuli were used during training of conditional discriminations and testing for emergent relations

Figure 1 shows the number of times each of the food items were incorrectly sorted for Conditions 1, 2, and 3, respectively. For all groups, a large number of items were categorized incorrectly. Figure 2 shows the details about the top five food items that were incorrectly sorted. Bananas were incorrectly sorted as "20–40" and "more than 40" carbohydrates per 100 g. Peanut butter was mainly sorted incorrectly as "more than 40" carbohydrates per 100 g. Potatoes were incorrectly sorted as "20–40" and "more than 40" carbohydrates per 100 g. White pepper was mainly sorted incorrectly as "less than 20" carbohydrates per 100 g. Garlic powder was sorted incorrectly as "less than 20" and "20–40" carbohydrates per 100 g but also as the “don’t know” option. The number of times the different food items were used in the conditional-discrimination training is shown in Figure 3.

Four participants, P13501, P13503, P13506, and P13512 were provided with an MTS task containing one food item previously sorted correctly. The remaining participants made enough incorrect sorting responses during the sorting test, so we could use 6 or 12 stimuli incorrectly sorted stimuli for participants in Conditions 1 and 2 and participants in Condition 3, respectively.

### Duration of the Experimental Sessions

Conditional-discrimination training and testing lasted from 15 to 24 min for the participants in the first two groups. For participants in the third group, the training and testing lasted from 45 to 84 min.

### Training Trials

For participants in Condition 1, the mean trials to the mastery criterion was 169 (150–240) (see Table 3). Participants in Condition 2 had a mean of 165 trials (150–240), and in Condition 3, the mean was 400 (240–640).

**Table 3:** Overview of the results

Condition 1				Condition 2				Condition 3			
P#	Trials	EQ	Post	P#	Trials	EQ	Post	P#	Trials	EQ	Post
13500	150	y/y	6/6	13510	240	y/y	6/6	13516	360	y/y	12/12
13501	180	y/y	6/6	13511	150	y/y	6/6	13519	660	y/y	12/12
13502	180	y/y	6/6	13512	120	y/y	6/6	13521	360	y/y	12/12
13503	180	y/y	6/6	13513	180	y/y	6/6	13522	420	y/y	12/12
13505	150	y/y	6/6	13514	150	y/y	6/6	13525	420	y/y	12/12
13507	150	y/y	6/6	13515	150	y/y	6/6	13518	469	n/n	2/2
13504	180	n/y	6/6	13523	150	y/y	6/6				
13506	180	n/y	6/6	13524	180	y/y	6/6				

*Note.* Column P# shows the participant number. Column EQ shows responding in accordance with stimulus equivalence (y) or not (no) in the two test blocks. Column Post shows the number of correct stimuli sorted in the two sorting, postclass formation tests

### Equivalence Class formation

All of the participants were exposed to two tests for emergent relations. Six out of the eight participants in Condition 1 responded in accordance with equivalence, and the remaining two participants responded in accordance with equivalence in the second test (see Table 3). All participants in Condition 2 responded in accordance with stimulus equivalence in both of the tests. In Condition 3, five of six participants responded in accordance with equivalence in both tests.

### Postclass Formation Sorting Tests

In the postclass formation sorting test, all the participants in Conditions 1 and 2 sorted the stimuli in accordance with the experimenter-defined classes (see Table 3). The one participant (P13518) who did not respond in accordance with equivalence did not sort the stimuli correctly in the sorting test. The participant sorted only two of the 12 stimuli correctly in both sorting tests. During the debriefing, the participant said that she knew the experimenter-defined classes, but she chose to respond in accordance with the classes she had sorted during the initial sorting task in the test for emergent relations.

## Discussion

### General Comments

In Phase 1, sorting and tailoring of stimuli, we included the “don’t know” option in Conditions 2 and 3 because the knowledge of the carbohydrates level was in general low for participants in Condition 1. The inclusion of the “don’t know” option was inspired by experiments that have included an additional option for giving a nonclass-based response in the test for emergent relations (e.g., Imam & Blanche, 2013). In the present experiment, the result of including “don’t know” showed that it was selected more in Condition 2 than in Condition 3, even if the number of stimuli in the sorting test was higher in Condition 3 than in Condition 1. The difference was mainly related to the performance of two participants in Condition 3 who used the “don’t know” option for several food items.

After the procedure based on stimulus-equivalence technology, 21 out of 22 participants responded in accordance with stimulus equivalence and sorted the stimuli according to the experimenter-defined classes. The greater number of training trials is the result of training on 12 conditional discriminations in Condition 3, while 6 conditional discriminations were trained in Conditions 1 and 2.

The goal of the present experiment was not to compare different methods for promoting knowledge about nutrition but rather to show how procedures based on stimulus-equivalence technology can be used to foster learning abilities within a specific context (e.g., de Abreu César & Moroz, 2018; Walker & Rehfeldt, 2012). The reason for using such technology is based on a substantial amount of research showing that training a few relations, will produce a larger number of relations that are not directly trained. Additionally, we wanted to show the effect of emergent relations not only after training a small number of conditional discriminations (Conditions 1 and 2) but also by including a greater number of conditional discriminations (Condition 3). Early in the history of research on stimulus equivalence, Sidman, Kirk, and Willson-Morris (1985) showed that it was possible by training 15 conditional relations to produce 60 emergent relations. Thus, the emergence of several untrained relations is in accordance with other experiments using procedures based on stimulus-equivalence technology or EBI (e.g., Fienup et al., 2016).

A necessary next step is the implementation of the laboratory procedure described in the present experiment in a classroom setting (see, e.g., Fienup et al., 2016) and a comparison of the effects of the procedure with traditional ways of teaching categories. Some studies have compared the effect of EBI and procedures based on other types of instructions, such as unstructured flash-card practice and have shown the superiority of the former (Zinn, Newland, & Ritchie, 2015).

Two of the participants in Condition 1 showed an increase in responding in accordance with experimenter-defined classes. Such an increase in responding according with equivalence class formation could be described as delayed emergence of equivalence. Such type of response pattern is reported in other experiments (e.g., Arntzen & Mensah, 2020; Arntzen & Nartey, 2018) and emphasizes the importance of having at least two test blocks to be able to observe changes during testing.

### Tailoring of Stimuli

In basic research on stimulus equivalence, experiments are arranged with conditional discriminations of abstract shapes that are arbitrarily related, and due to the training of conditional discriminations and testing of emergent relations the stimuli in the set are partitioned into different classes (e.g., Sidman & Tailby, 1982). However, in experiments characterized as stimulus-equivalence technology or EBI, the stimuli used are often meaningful stimuli and definitely not abstract shapes (e.g., Fienup et al., 2010; Varelas & Fields, 2017), and it could be that some of the stimuli are already part of the trained stimulus classes. Thus, individually tailoring the stimuli, as was done in the present experiment, is essential. Hence, the stimuli used for each participant were tailored based on the results from the sorting test. This type of arrangement for tailoring of the stimuli used in the present experiment is in accordance with other experiments (Nastally et al., 2010), and tailored nutritional education has been highlighted by other researchers (Oenema, Brug, & Lechner, 2001).

### Correspondence of Test Formats

The findings in the present experiment showed a high correspondence between equivalence-class formation in the MTS test and sorting according to experimenter-defined classes. These results are in accordance with a series of experiments (e.g., Arntzen, Granmo, & Fields, 2017). Sorting is an alternative measurement, especially in applied settings, because it is less time-consuming than the MTS test and easy to administer (e.g., Rustad Bevolden & Arntzen, 2018) and can also be useful in studies involving procedures based on stimulus-equivalence technology or EBI.

## Limitations and Further Experiments

The design in the present study is only a demonstration and future research should use either a between-subjects or within-subject design. For four of the participants, one of the stimuli employed in the conditional-discrimination training was a correctly sorted food item. For P13512, who had been trained on 12 conditional discriminations, this was not as influential for the three other participants (P13501, P13503, and P13506), who had been trained on six conditional discriminations. It is important to note that the results of these four participants did not differ from the other participants with respect to performance on the MTS tests. Additionally, three of the participants, P13501, P13503, and P13506, had more trials to mastery criterion than the average for the group, whereas P131512 had a fewer trials than the average. On the other hand, Nastally et al. (2010) do not report how many stimuli or how many participants performed the MTS tasks with stimuli they had sorted correctly during Phase 1.

The carbohydrate values differ for some of the food items depending on if they are raw or cooked. It is important to emphasize that even if this information was not given for all food items it did not influence the results in establishing the conditional discriminations. However, in further research, this should be specified in more detail.

Nastally et al. (2010) included some participants with a high BMI, which was not part of the aim in the present experiment. However, there is a need for relating knowledge about nutrition regarding carbohydrates, as in the present experiments with an arrangement of diets to reduce weight or for type 2 diabetes management as emphasized in other types of research (e.g., Foster, 2006; Tay et al., 2015).

In further experiments, the postclass formation sorting test should be arranged before the MTS test to explore the correspondence between the two test formats (see, e.g., Arntzen et al., 2017). If the sorting test after training shows partitioning of classes, such tests could be beneficial in applied settings. Additionally, future studies should include follow-up tests for evaluating the long-term effects of MTS training.

## Summary and Implications

The present experiment replicated the findings from Nastally et al. (2010). It showed the effectiveness of using a tailored selection of stimuli used in a computerized procedure to produce classes of stimuli of food items within the same range of carbohydrate values that were not explicitly trained. We would argue that tailoring stimuli should be an essential feature of EBI research.

There are several implications of this type of stimulus-equivalence technology study, regarding efficiency and efficacy. First, the duration of the experimental sessions shows that the procedure is not time consuming but is still effective in training knowledge about carbohydrate values for different food items. The difference in duration between Conditions 1 and 2, in comparison to Condition 3, is related to the number of conditional discriminations trained and tested. Second, the procedure based on stimulus-equivalence technology efficiently improved knowledge about the nutritional content in a variety of food items.

It is important to distinguish between slow and fast carbohydrates. A variety of fruits and vegetables, as well as grains such as whole wheat, are examples of slow carbohydrates. Fast carbohydrates are, for example, found in drinks and refined grains such as white rice and those in cornbread, white bread, grits, and couscous. Characteristically, fast carbohydrates will be low in fiber, whereas slow carbohydrates are fiber-rich and healthy nutrients. Blood sugar rises faster after consumption of fast carbohydrates compared to slow carbohydrates, and the differences in blood sugar level will influence how soon you will feel hungry after having eaten (e.g., Chandler-Laney et al., 2014). Increasing the intake of slow carbohydrates while minimizing or eliminating fast carbohydrates is key to improving health; thus, it is essential to teach participants in stimulus-equivalence technology research projects to differentiate between slow and fast types of carbohydrates.
